# Unidirectional Drug Delivery and Responsive Release Guided by Nanofunnel-Shaped Heterojunction

**DOI:** 10.1021/acs.nanolett.5c00617

**Published:** 2025-05-05

**Authors:** Jun Luo, Changxiong Huang, Zhenyu Liao, Xinyao Ma, Ting Si, Huan Chen, Zhen Li, Jun Fan

**Affiliations:** † Department of Materials Science and Engineering, 53025 City University of Hong Kong, Hong Kong, 999077, China; ‡ Department of Physics, 53025 City University of Hong Kong, Hong Kong, 999077, China; § School of Materials Science and Engineering, China University of Petroleum (East China), Qingdao 266580, China; ∥ Center for Advanced Nuclear Safety and Sustainable Development, 53025 City University of Hong Kong, Hong Kong, 999077, China; ⊥ Department of Mechanical Engineering, 53025 City University of Hong Kong, 83 Tat Chee Avenue, Kowloon, Hong Kong, 999077, China

**Keywords:** unidirectional drug delivery, nanocone, boron nitride, graphene, curvature, molecular dynamics simulations

## Abstract

Drug resistance often involves preventing drug entry or expelling drugs from cells, severely affecting the therapeutic effect. We propose nanofunnel-shaped devices by pairing truncated graphene/boron nitride nanocones and nanotubes using curvature gradients and material properties to modulate energy barriers for unidirectional drug delivery. Molecular dynamics simulations demonstrate spontaneous delivery across models (Δ*G* = −14.14 to −27.87 kcal·mol^–1^ for C_∨_-C_||_, BN_∨_-BN_||_, C_∨_-BN_||_). The potential of mean force calculations reveal energy barriers scale as Δ*G* ∝ 1/*R* ^2^, with BN nanotubes showing 20–30% higher barriers (e.g., −19.63 vs −14.14 kcal·mol^–1^ for graphene) due to stronger van der Waals interactions. In the BN_∨_-C_||_ model, increasing the tube radius (9.59 to 20.34 Å) or decreasing the cone angle (180–60°) can flip Δ*G*, enabling bidirectional control. This curvature–material synergy bypasses efflux mechanisms, offering a tunable platform to combat drug resistance and enhance therapeutic precision.

## Graphical abstract

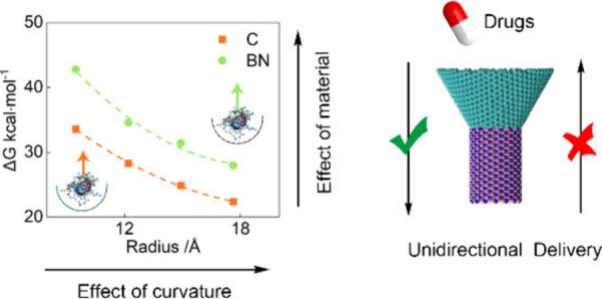

Mass transfer through nanoscale channels is a fundamental process in nature and living systems, underpinning critical advancements in modern science and engineering.^1–3^ Research into this nanoscale transport phenomenon is essential for advancing fields such as drug delivery, chemical reactions, and nanopore sequencing.^4–7^ In oncology, effective drug delivery remains a pressing challenge, as conventional chemotherapy, targeted therapies, and immunotherapies often suffer from nonspecific cytotoxicity and drug resistance.^8–13^ Drug resistance is the main cause of treatment failure. In addition to directly preventing drugs from entering diseased cells, it also reduces intracellular drug concentration through mechanisms such as extracellular efflux pumps, thus accelerating cancer recurrence and patient death.^14–19^ For example, ATP-binding cassette transporters actively expel chemotherapeutic agents like doxorubicin, undermining therapeutic efficacy.^20^ These limitations highlight the urgent need for innovative drug delivery systems that can overcome direct resistance and drug efflux.

To address these challenges, unidirectional delivery systems have been proposed to prevent drug backflow, sustain therapeutic levels, and enhance the precision of drug delivery, thereby mitigating systemic toxicity. Natural channel proteins that regulate intracellular molecular transport widely exist in the human body, but their complexity and instability make them difficult to use for scalable experimental or therapeutic purposes.^21–23^ To address this, artificial nanochannels, such as carbon nanotubes (CNTs), have emerged as biomimetic alternatives.^24,25^ Studies have demonstrated that CNTs can integrate into lipid membranes to form stable transmembrane porins, facilitating the transport of water, ions, and small molecules.^24,26,27^ Using CNTs, drug encapsulation can be achieved, but lacked directional control or tunability; while research on 2D materials in drug delivery has mainly involved single, static adsorption, heterostructures have rarely been explored.^28–31^ A critical shortcoming of these CNT systems is their uniform tubular structure, which permits bidirectional transport and risks drug leakage, thus failing to sustain therapeutic levels in target cells.^32,33^ Recent studies suggest that varying nanotube curvatures can affect drug interactions,^34,35^ and designing a curvature gradient along the CNT could achieve unidirectional delivery. Furthermore, with the development of nanotechnology, the preparation of various nanomaterials is becoming more and more mature, including the preparation of nanoparticles, nanorods, nanoclusters, nanosheets, nano-heterojunctions, and so on.^36–42^ Different nanomaterials possess unique physiochemical properties and interactions with molecules. By the formation of heterostructures, these properties can be combined to enhance functionality. Here, we introduce a novel nanofunnel-shaped platform that couples truncated nanocones with nanotubes to achieve controlled unidirectional drug transport. By leveraging a curvature gradient at the cone–tube junction, our design prevents backflow, addressing the leakage problem inherent in uniform CNTs. Furthermore, by integrating different two-dimensional materials into the cone and tube components, we exploit their unique physicochemical properties, such as surface interactions, to optimize the system for specific drug delivery needs.^43,44^ This heterostructure approach builds on prior curvature-driven transport studies while offering unprecedented tunability, distinguishing our work from existing CNT-based platforms.

In this study, we refine CNT-based nanofunnel systems and assess their drug delivery performance using molecular dynamics simulations. By testing three different drug types, we demonstrate the platform’s universality across diverse therapeutic agents. Thermodynamic analyses confirm that curvature-driven unidirectional transport reduces backflow, advancing membrane-integrated delivery systems with enhanced structural and material tunability. This approach offers a promising strategy to overcome drug resistance and improve the therapeutic precision.

Considering the unique properties of nanotubes, we optimized their structure to enable unidirectional drug delivery. As shown in Figure 1(a), four models, C_∨_-C_||_, BN_∨_-BN_||_, C_∨_-BN_||_, and BN_∨_-C_||_, were constructed with a “truncated nanocone” for drug capture and a “nanotube” for storage and release. We tested three drug types (peptide 2OVN, nucleic acid siRNA, and small molecule DOX). Simulations assessed drug delivery across all four models, with drugs initially positioned at the cone’s entry. Figure 1(b) presents the drug morphology during the simulation, using 2OVN as an example, and shows snapshots at various stages across the four models. Figure 1(c) tracks the center-of-mass (CoM) position of drugs along the *Z*-axis over the simulation time. Notably, all three drug types exhibited consistent delivery outcomes across the four models. Specifically, unidirectional delivery was successfully achieved in the C_∨_-C_||_, BN_∨_-BN_||_, and C_∨_-BN_||_ models. However, the BN_∨_-C_||_ model exhibited a failure in drug delivery. This failure likely reflects a mismatch between BN’s strong drug affinity in the cone and graphene’s weaker pull in the tube, creating an energy barrier. The material composition significantly influences the interactions between the drugs and the delivery devices. Compared with conventional CNTs, our curvature–gradient design achieves unidirectional delivery, directly addressing the critical leakage problem in transmembrane drug delivery systems. In the context of the Figure 1(c3) C_∨_-C_||_ model, it is observed that while the drug can be effectively delivered into the tube, there is a tendency for it to remain at the junction. This behavior likely stems from a low free energy at the junction, which is discussed later. These results highlight the material composition as a critical parameter. BN’s stronger interaction in C_∨_-BN_||_ enhances delivery speed. In contrast, BN_∨_-C_||_’s failure underscores the need for balanced cone–tube interactions, setting the stage for structure optimization.

**Figure 1:**
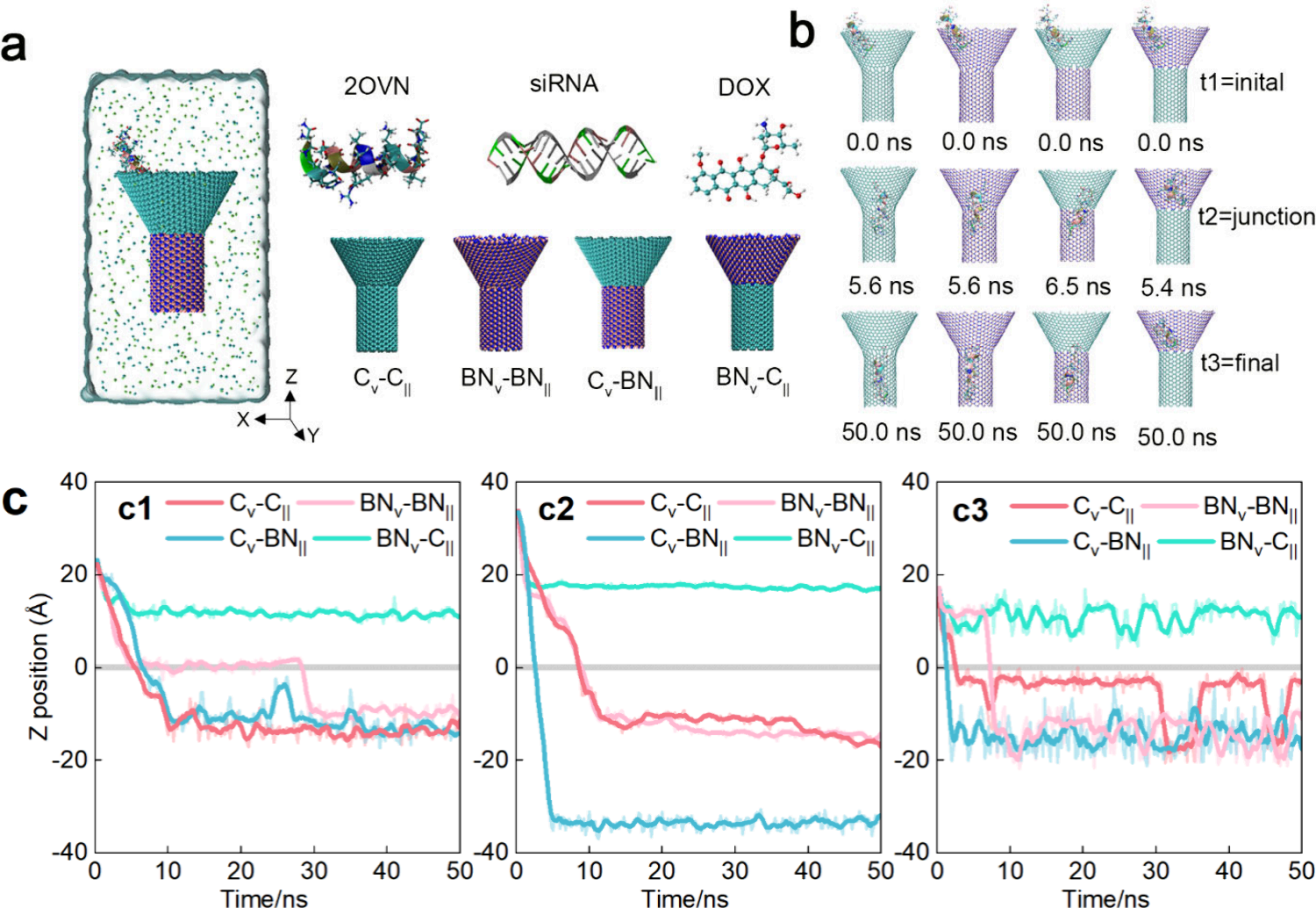
Drug delivery in different nanofunnel-shaped heterojunction channels. (a) The simulated models are a combination of three different types of drugs (peptide 2OVN, nucleic acid siRNA, small molecule drug DOX) and four delivery models, C_∨_-C_||_, BN_∨_-BN_||_, C_∨_-BN_||_, and BN_∨_-C_||_ (each model consists of two parts: cone and tube; C or BN represents that the material of this part is graphene or boron nitride). In the heterojunction models, the blue, pink, and cyan spheres represent the nitrogen, boron, and carbon atoms, respectively. (b) Taking peptide 2OVN as an example, snapshots of the drug delivery process from cone to tube in four different channel models are shown. t1, t2, and t3 represent the initial, junction, and final moment of the simulation, respectively. Water molecules are not displayed for clarity. (c) The position of the center-of-mass (CoM) of (a1) 2OVN, (a2) siRNA, and (a3) DOX on the *Z*-axis as a function of simulated time. Position 0 of the *Z*-axis corresponds to the junction of the cone and tube.

To assess material impacts, we measured the average contact number (within 4 Å) between drugs and model surfaces over the last 10 ns in Figure 2(a). For homogeneous models C_∨_-C_||_ and BN_∨_-BN_||_, the BN_∨_-BN_||_ model exhibited stronger interactions, with higher average contact numbers for 2OVN (112 ± 3.5 vs 88 ± 6.1) and siRNA (381 ± 11.5 vs 327 ± 5.4) compared to C_∨_-C_||_. This arises from BN’s stronger van der Waals interactions compared to graphene, enhancing drug adhesion. These findings align with prior studies.^45,46^ The stronger interaction between BN and drug molecules also explains the fastest drug delivery under the C_∨_-BN_||_ model in Figure 1(c). For DOX, due to its planar structure and smaller size, it exhibited stable adsorption across all models with negligible contact variation.

**Figure 2:**
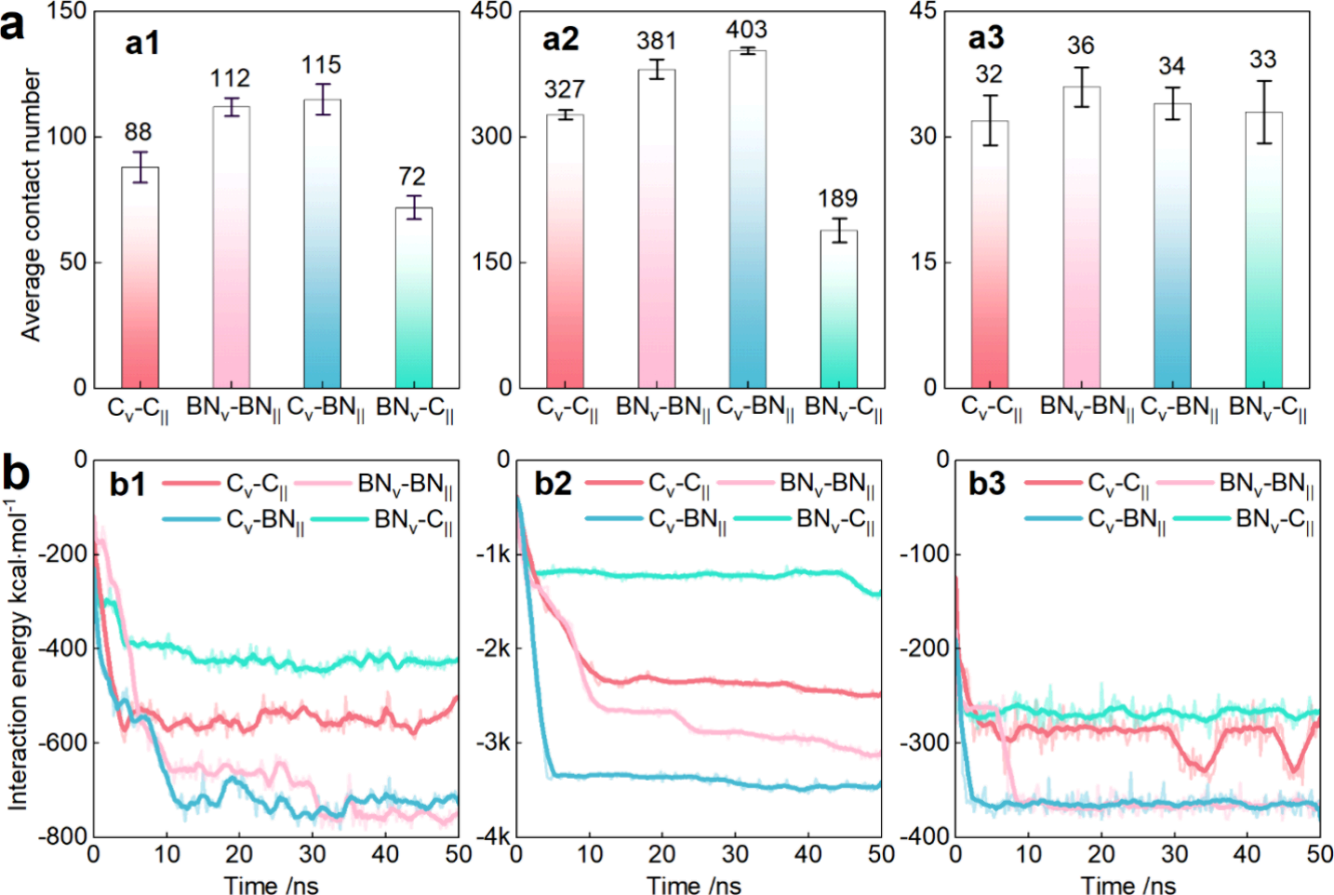
Analysis of the influence of different material types on drug encapsulation. (a) The average contact number between channel models and the drugs (a1) 2OVN, (a2) siRNA, and (a3) DOX (within 4 Å of the channel models). (b) Interaction energy between the (b1) 2OVN, (b2) siRNA, (b3) DOX, and the channel models as a function of simulation time.

Previous studies identify van der Waals interactions as the primary driving force of drug encapsulation in carbon nanotubes.^47,48^ Accordingly, we analyzed interaction energies between the three different types of drugs and the models. As can be seen from Figure 2(b), in successful delivery models (C_∨_-C_||_, BN_∨_-BN_||_, and C_∨_-BN_||_), the interaction energy increased as drugs moved into the tube and stabilized thereafter. For Figure 2(b1) 2OVN, BN_∨_-BN_||_ and C_∨_-BN_||_ showed energy increases of 402.69 kcal·mol^–1^ and 555.44 kcal·mol^–1^, respectively. The increased interaction energy (around 79.93 kcal·mol^–1^) in the C_∨_-BN_||_ model primarily results from the cone’s different material, as tube interactions remain comparable. Comparing C_∨_-C_||_ (333.14 kcal·mol^–1^) and C_∨_-BN_||_, since the cone material is the same, the initial interaction energy for both models is about 200 kcal·mol^–1^, so the increased interaction (around 222.30 kcal·mol^–1^) is mainly due to the different tube material. For siRNA and DOX, BN_∨_-BN_||_ exhibits stronger interactions (−3461.11 vs −3050.19 kcal·mol^–1^ for siRNA; −365.23 vs −330.10 kcal·mol^–1^ for DOX) than C_∨_-C_||._ Both contact numbers and energy analyses confirm BN’s stronger drug interaction, correlating with C_∨_-BN_||_’s enhanced delivery efficiency. These results highlight the material composition as a key parameter enhancing the delivery efficiency. The observed hierarchy of material-dependent interactions (BN > C), extending these insights to directional transport control, demonstrates the unique advantages of two-dimensional material hybridization in creating energy landscape gradients, an ability lacking in single-material systems.

To assess thermodynamic favorability, we calculated the potential of mean force (PMF) for drug delivery from the cone to tube (Figure 3). Given the consistent delivery outcomes for siRNA and 2OVN, we calculated the PMF only for 2OVN and DOX. For 2OVN in Figure 3(a), whose size matches the tube’s, the PMF decreases as 2OVN enters the tube from the cone in the C_∨_-C_||_, BN_∨_-BN_||_, and C_∨_-BN_||_ models, confirming the delivery is a spontaneous process and is consistent with the simulation results in Figure 2(a). Additionally, in Figure 3(c), the free energy barriers of these models are, from small to large, Δ*G*_C_∨_–C_||__ (−14.14 kcal·mol^–1^), Δ*G*_BN_∨_–BN_||__ (−19.63 kcal·mol^–1^), and Δ*G*_C_∨_–BN_||__ (−27.87 kcal·mol^–1^), aligning with material effects, which is a key parameter. The BN_∨_-C_||_ model, with the smallest Δ*G* (−10.94 kcal·mol^–1^), exhibits a 3 kcal·mol^–1^ energy barrier at ξ ≈ 0.5 nm (Figure 3a, 3c), explaining the lack of spontaneous delivery in simulations. Size constraints likely required conformational adjustments for tube entry, while BN_∨_-C_||_’s smaller free energy difference delayed the process within simulation time scales. For DOX, which is smaller than the tube, PMF profiles confirmed spontaneous delivery (Δ*G* < 0) in C_∨_-C_||_, BN_∨_-BN_||_, and C_∨_-BN_||_ models. C_∨_-C_||_ shows a low free energy minimum at the junction (ξ = 0 nm, Figure 3(b)), aligning with observed DOX accumulation (Figure 2(a3)). In contrast, BN_∨_-C_||_ showed Δ*G* > 0 (4.48 kcal·mol^–1^), indicating nonspontaneous delivery. These results, aligned with simulations, demonstrate material-dependent energy landscapes driving the delivery efficiency. Unlike passive diffusion in traditional nanotubes, our heterojunctions leverage material-specific energy landscapes, enhancing control over delivery spontaneity and addressing gaps in directional delivery.

**Figure 3:**
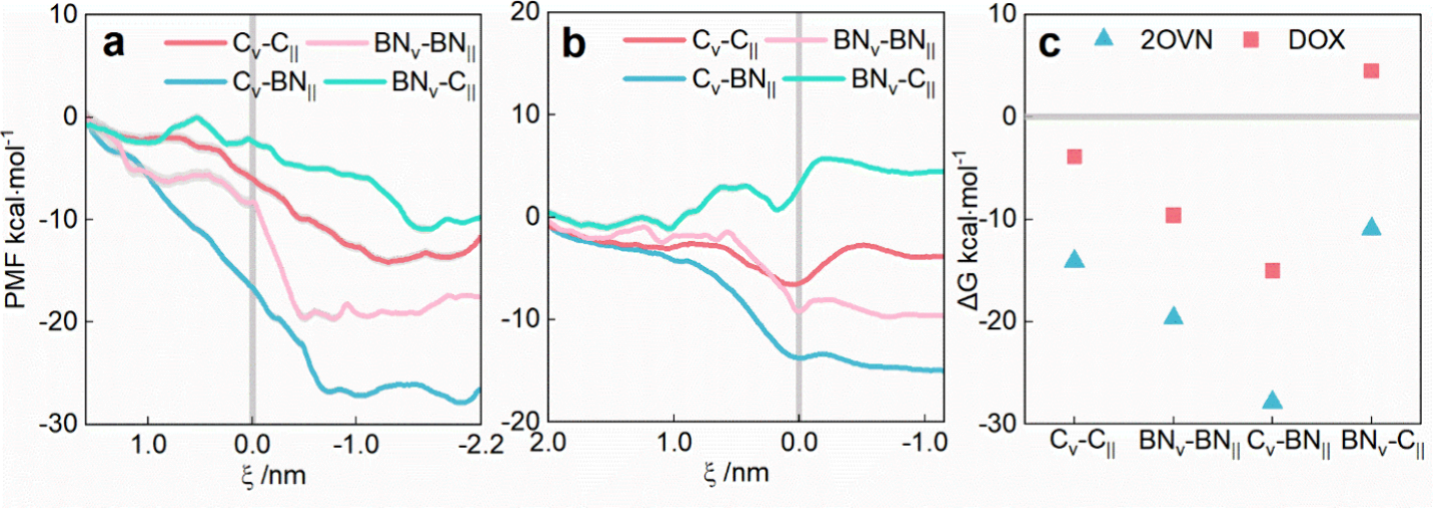
PMF as a function of the CoM distance in the *Z*-axis between drugs (a) 2OVN and (b) DOX and the tube of four channel models. The free energy profiles can be read from left to right corresponding to the insertion processes of the drugs. Position 0 of the *X*-axis corresponds to the junction of the cone and tube. (c) Summary of figures (a) and (b), corresponding to the difference in free energy of the drug in the tube and cone.

In our models, drugs undergo a “tug-of-war” between the cone and tube, enabling delivery control by adjusting their relative strengths. The strength of each component can be adjusted through surface curvature, which governs drug–nanomaterial interactions. Here, we calculated the PMF to assess the detachment of a 2OVN molecule from a half-tube with varying curvatures. Curvature, defined as κ = 1/*R* (*R* is the tube radius), decreases with increasing tube radius. We examined four armchair nanotubes with corresponding diameters *R* = 9.49, 12.20, 14.92, and 17.63 Å for graphene and 9.69, 12.46, 15.24, and 18.00 Å for boron nitride. BN nanotubes have slightly larger radii than graphene due to BN’s longer bond length. To eliminate size effects, we maintained a constant atom count across different curvatures. The detailed configuration can be seen in . Figure 4(a1, a2) show that as 2OVN detaches, PMF values increase before plateauing when no further interaction occurs. This trend is consistent for both graphene and BN. As tube curvature decreases (*R* increases), the free energy barrier (Δ*G*) required to detach the 2OVN molecule decreases, reflecting weaker confinement in larger tubes due to reduced surface curvature and revealing a positive correlation between curvature and drug–nanomaterial interactions. For a more intuitive depiction of the impact of curvature and materials, Figure 4(a3) displays the Δ*G* values for two different materials at four distinct tube radii, with the dashed line representing the fitted data (detailed data in ). This curvature–drug interaction scales as Δ*G* ∝ 1/*R* ^2^, offering a predictive tool for design optimization. Furthermore, at identical curvatures, BN’s higher Δ*G* indicates greater drug affinity than graphene. Thus, for the four channel models, despite fixed material types due to the influence of curvature, we may be able to control the delivery by adjusting the curvature of the cone or tube. Curvature emerges as a critical parameter for tuning the delivery yield, offering a novel lever beyond material choice, unlike static systems in prior studies.

**Figure 4:**
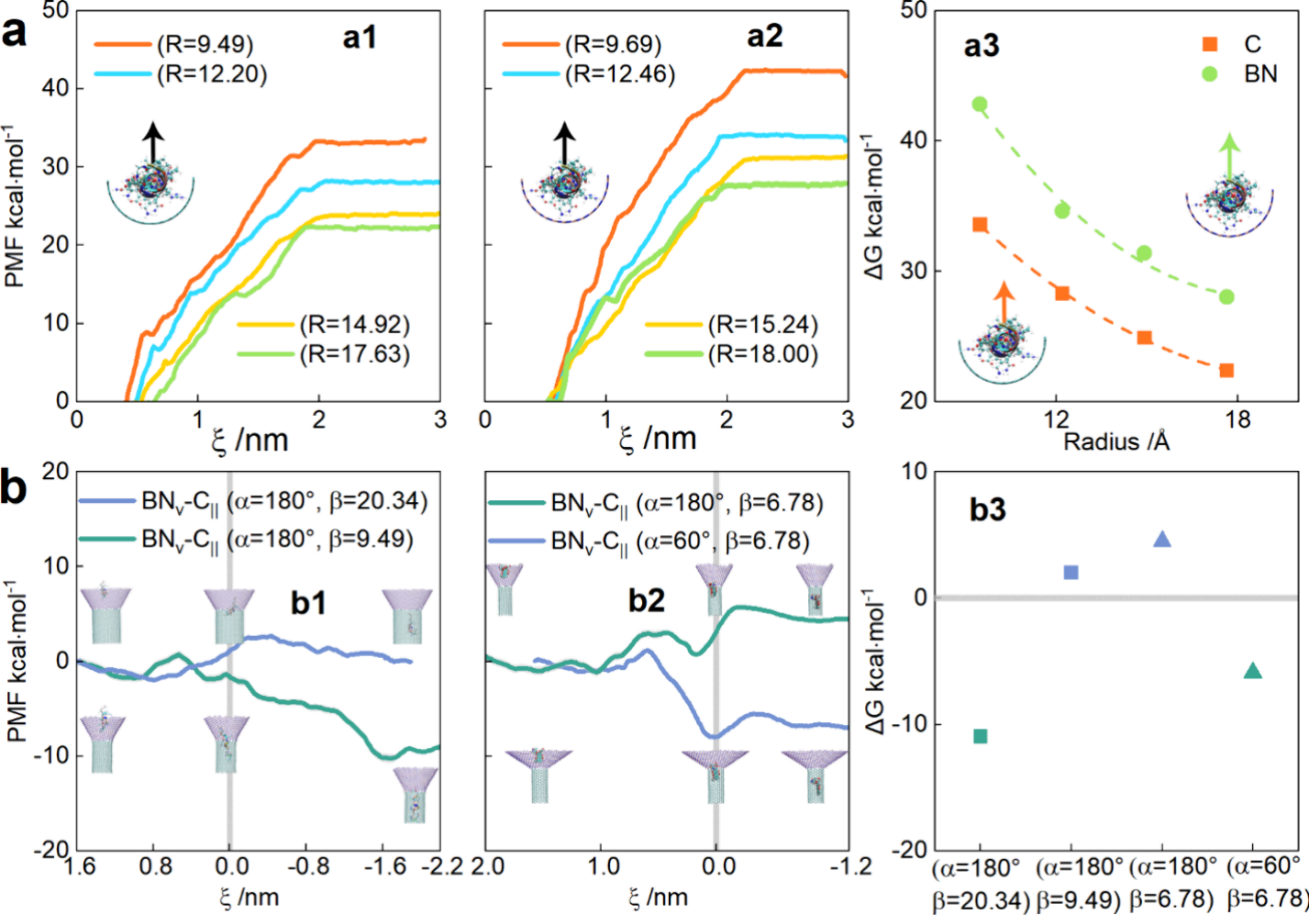
PMF as a function of the CoM distance between drugs and tubes. (a) Process of the drug moving away from the surface of the half tube (a1) C or (a2) BN. *R* represents the radius of the half tube. (a3) The free energy barrier for pulling drugs away from the half tube surfaces of C or BN with different radii; the dashed line is the fitting curve. (b) PMF profiles for adjusting the curvature of the channel models. (b1) Adjusting the radius of the tube to change the curvature to regulate drug delivery. (b2) Adjusting the helix angle of the cone to change the curvature to regulate drug delivery. α represents the disclination angle of the cone; β represents the radius of the tube. Position 0 of the *X*-axis corresponds to the junction of the cone and tube. (b3) Summary of figures (b1) and (b2), corresponding to the difference in free energy of the drug in the tube and cone.

Combined with the results we obtained in Figure 4(a), we observed the unique behavior of the BN_∨_-C_||_ model. Unlike the other three models where the tube always dominates, the cone and tube in the BN_∨_-C_||_ model dominate in terms of material and curvature, respectively. Unlike the tube-dominant C_∨_-C_||_, BN_∨_-BN_||_, and C_∨_-BN_||_ models, BN_∨_-C_||_ features a cone (BN) and tube (C) competing via material and curvature, respectively. Since the material of the BN_∨_-C_||_ model is determined, we can control the delivery by adjusting the curvature. If the curvature of both the cone and tube is minimized (e.g., approaching a flat nanosheet), geometric effects diminish and material properties become the dominant factor. Thus, decreasing tube curvature weakens its influence, enhancing the cone’s competitiveness despite its curvature reduction. From Figure 4(b1, b3), we can see that with a fixed cone disclination angle, increasing the tube radius from α = 180°, β = 9.59 (Δ*G*_BN_∨_–C_||__ = −10.94 kcal·mol^–1^) to α = 180°, β = 20.34 (Δ*G*_BN_∨_–C_||__ = 2.05 kcal·mol^–1^) flips the energy barrier from negative to positive. This reversal occurs because larger tubes weaken the tube’s pull (lower κ), allowing BN’s strong cone affinity to dominate. This reversal indicates tunable delivery directionality in the BN_∨_-C_||_ model under these two different tube curvatures. The snapshots in the figure correspond to the positions of the drug at the initial, junction, and final moments. Likewise, reducing cone curvature by decreasing its disclination angle (180° → 60°) under constant β flips Δ*G* from positive (4.48 kcal·mol^–1^) to negative (−5.91 kcal·mol^–1^), enabling controlled delivery in Figure 4(b2, b3). A flatter cone (lower α) reduces its curvature, decreasing drug adhesion and favoring tube-directed delivery. This dual curvature modulation (tube or cone) allows programmable drug transport in the BN_∨_-C_||_ model. Inspired by this adjustability, we simulated reverse delivery (tube-to-cone) in cone-dominated configurations (), confirming the bidirectional control potential. Bidirectional control in BN_∨_-C_||_ by curvature modulation represents a shift from a static transfer system. Unlike nanomaterials, which require a pH or temperature response to environmental changes, our geometric tuning allows for programmable transmission by using fixed material components.

In drug delivery through transmembrane channels, releasing drugs from the channel is just as important as their entry. Our results show successful unidirectional drug transport within channel models, with drugs retained in nanotubes absent of additional stimuli. To facilitate drug release, additional driving forces are necessary. Using protonated DOX (DOXH) as an example, we can apply an external electric field as a driving force due to DOXH’s positive charge. Figure 5 presents the process of DOXH release under an electric field stimulation. In this simulation model, the C_∨_-BN_||_ is embedded in the 1-palmitoyl-2-oleoylsn-glycerol-3-phosphocholine (POPC) lipid membrane, and a DOXH molecule layer is placed 5 Å above the C_∨_-BN_||_. We simulated 50 ns without an electric field, followed by 30 ns with a 0.15 V/nm field along the *Z*-axis. As seen from the figure, DOXH spontaneously enters the tube without an electric field and then stays at the bottom. Upon electric field application, DOXH begins to gradually detach from the tube and is finally released into the aqueous solution. The field overcomes the energy barrier at the exit of the tube, shifting the energy landscape to favor the release. For a more complete view of the model, we also show snapshots with no electric field, *E_z_* = 0.00 V/nm (DOXH filled with tube), and with an electric field *E_z_* = 0.15 V/nm (DOXH released from the tube). Thus, electric fields provide a reliable method for the controlled release of charged drugs from the nanotubes. This electric field approach, unlike passive release methods, which risk incomplete release, offers precise temporal control, addressing a key limitation in nanotube-based delivery systems.

**Figure 5:**
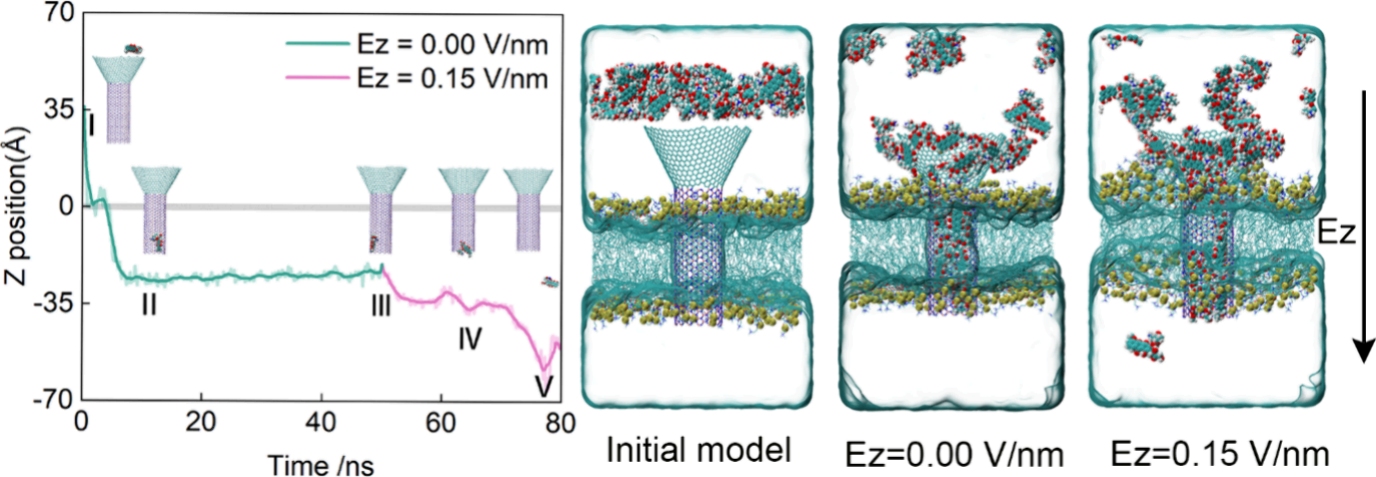
Drug release is driven by an electric field. The position of CoM of a released DOXH on the *Z*-axis as a function of simulated time (left). The insets I, II, and III correspond to the positions of DOXH at the beginning, the bottom of the tube, and the end of the simulation time with *E*_*z*_ = 0.00 V/nm. IV and V correspond to the positions of DOXH detached from the tube and in the aqueous solution with *E*_*z*_ = 0.15 V/nm. For the sake of clarity, only the DOXH and channel models are shown. We also present the snapshot of the initial model, the drug encapsulation model without an electric field, and the drug release model driven by an electric field on the right. The tan spheres represent P8 atoms in POPC.

We developed 3D nanofunnel heterojunctions using 2D materials for unidirectional drug delivery. Our design leverages two insights: (1) drug–nanotube interactions scale with curvature and (2) graphene and BN’s structural similarity enables hybrid structures. These insights yielded four channel models (C_∨_-C_||_, BN_∨_-BN_||_, C_∨_-BN_||_, and BN_∨_-C_||_), with transport directionality controlled by a cone–tube “tug-of-war”. In C_∨_-C_||_ and BN_∨_-BN_||_, tube curvature drives the delivery. For the C_∨_-BN_||_ hybrid, BN’s material and curvature synergy enhance delivery. Conversely, the BN_∨_-C_||_ model offers tunability, balancing cone material, and tube curvature through geometric changes. Adjusting tube radius or cone disclination angle shifts dominance to the cone or tube. Additionally, simulations confirmed reverse delivery from the cone to tube and electric-field-driven DOXH release in C_∨_-BN_||_, showcasing programmability. Compared to prior 2D material studies, our heterojunctions uniquely combine material and curvature effects for enhanced unidirectional control. This work advances programmable delivery systems, addressing a gap in directional control. However, challenges like membrane insertion and universal drug release methods remain.

## Supplementary Material













## Data Availability

Data will be made available on reasonable request.

## Notes

Methods section, Section S1: Summary of simulations, Section S2: Nanotube models with different curvatures, Section S3: Nanotube models with different curvatures and the corresponding free energy barrier to pull 2OVN away from the nanotube, and Section S4: Reverse drug delivery from tube to cone (PDF)
Supporting Movie SM1: Simulation of unidirectional drug delivery of 2OVN (MP4)
Supporting Movie SM2: Simulation of unidirectional drug delivery of siRNA (MP4)
Supporting Movie SM3: Simulation of unidirectional drug delivery of DOX (MP4)
Supporting Movie SM4: Reverse drug delivery (MP4)
Supporting Movie SM5: Release of DOXH with an electric field (MP4)
