# Supplementary material for: Unidirectional Drug Delivery and Responsive Release Guided by Nanofunnel-Shaped Heterojunction
Source: Nano Lett. 2025 May 5;25(19):7853–9. doi: 10.1021/acs.nanolett.5c00617 (PMC12713770; doi:10.1021/acs.nanolett.5c00617)
Supplement: Supplementary file 1 [file nl5c00617_si_001.pdf]

Supporting Information for

Unidirectional Drug Delivery and Responsive Release guided by Nanofunnel-shaped Heterojunction

Jun Luo [1], Changxiong Huang [1], Zhenyu Liao [1], Xinyao Ma [1], Ting Si,[2], Huan Chen [1], Zhen Li [3,] , Jun Fan [1,4,5,*]*

[1] Department of Materials Science and Engineering, City University of Hong Kong, Hong Kong, 999077, China

[2] Department of Physics, City University of Hong Kong, Hong Kong, 999077, China

[3] School of Materials Science and Engineering, China University of Petroleum (East China), Qingdao 266580, China

[4] Center for Advanced Nuclear Safety and Sustainable Development, City University of Hong Kong, Hong Kong, 999077, China

[5] Department of Mechanical Engineering, City University of Hong Kong, 83 Tat Chee Avenue, Kowloon, Hong Kong 999077, China

*Email address: junfan@cityu.edu.hk, zhenli@upc.edu.cn

Methods section

Model Construction. For channel models, they are all composed of a half-nanocone combined with a nanotube. We built nanocones and nanotubes using the Nanotube Modeler software.[1] The disclination angle of the nanocone is 180°. Then, we cut the nanocone at a position where the diameter of the nanocone cross section is equal to the diameter of the nanotube. The height

of the half-nanocone we retained is 2nm. As for nanotubes, the length and radius we choose are mainly based on the size of the drugs. For 2OVN, (14, 14) nanotube was selected, with a radius of 9.49 Å, and a length of 30 Å. For siRNA, (20, 20) nanotube was selected, with a radius of 13.56 Å, and a length of 70 Å. For DOX, (10, 10) nanotube was selected, with a radius of 6.78 Å, and length of 30 Å. For drugs, three different types of drugs were selected. The first is a polypeptide, from the Protein Data Bank (PDB), entry code 2OVN,[2] with a sequence of NYHLENEVARLKKLCGE. The second is a nucleic acid molecule. We selected a siRNA sequence that is employed to silence full-length hepatitis C virus particles experimentally.[3] We obtained the siRNA sequence from VIRsiRNAdb which is a curated collection of viral siRNA/shRNA that has been validated experimentally.[4] For this siRNA, the sense sequence is GGCUGAUCAACACACACGGCA. The last one is a small drug molecule, we chose Doxorubicin (DOX, $C_{27}H_{29}NO_{11}$), a broad-spectrum anti-tumor anthracycline antibiotic. For the lipid membrane, we simulated 256 1-palmitoyl-2-oleoylsn-glycerol-3-phosphocholine (POPC) lipids (128 in each leaflet) for 300 ns at 310 K in the NPT ensemble to get the fully relaxed lipid bilayer.

Simulation Setup. The molecular dynamics (MD) simulation was carried out with the GROMACS software package[5]. The CHARMM36 force field was used for polypeptide and siRNA,[6], and the SPC/E model was used for water molecules.[7] The Lennard-Jones (LJ) parameters for carbon, nitrogen, and boron atoms were adopted from previous studies.[8,9] The force field parameters for the drug molecules DOX are generated by using the SwissParam web server.[10] For POPC, we adopted the extensively validated Berger lipid force field.[11-14] In the simulation, the covalent bonds involving hydrogen atoms were constrained using the LINCS

algorithm,[15] and the time step was 1fs. The long-range electrostatic interactions were treated using the particle mesh Ewald (PME) method.[16,17] The van der Waals (vdW) interactions were calculated with a cutoff distance of 1.2 nm. The temperature was kept constant at 310 K using a V-rescale thermostat,[18] and the pressure was set at 1 bar using a Parrinello−Rahman barostat.[19] After the energy minimization, the systems were equilibrated in NVT and NPT ensembles for 4 ns. Then, 50 ns production simulations were conducted in the NPT ensemble. In the drug release part, after 50 ns simulation, an electric field along the z-axis, Ez = 0.15 V/nm, is applied in the model to let the DOXH release from the tube.

Potential of Mean Force (PMF) Calculation. The PMF profiles were calculated with the umbrella sampling method[20-22] and the biased probability distributions were collected and unbiased with an implementation of WHAM[23] in GROMACS, using 200 bins and a tolerance of 0.0001. The interval of umbrella sampling windows is 1 Å. In each window, the center of mass distance in the z-axis between the drug and tube was restrained with a harmonic force constant of 2000 $kJ\cdot mol^{-1}\cdot nm^{-2}$. Each window was simulated for 25 ns, and the PMF results of the last 15 ns were used for sampling.

Supplementary Figure and Tables

S1-1. Summary of Simulations

Table S1 Summary of simulation models and simulation time. (simulations of the potential of mean force are not included)

	Model	Cone angle	Tube radius (Å)	Simulation time (ns)	Total time (ns)
Drug delivery	2OVN (C/C, BN/BN, C/BN, BN/C)	180°	9.49	50 × 6 (repeats) × 4(models)	1200 ns
	siRNA (C/C, BN/BN, C/BN,BN/C)	180°	13.56	50 × 6 (repeats) × 4(models)	1200 ns
	DOX (C/C, BN/BN, C/BN, BN/C)	180°	6.78	50 × 6 (repeats) × 4(models)	1200 ns
Reverse drug delivery	DOX BN/C	180°	6.78	50 × 3 (repeats)	150 ns
Drug release	DOXH C/BN	180°	9.49	50 (Ez = 0.00V/nm) 30 (Ez = 0.15V/nm)	80 ns
PMF	2OVN DOX			25 × 35 (windows) × 10(models)	8750 ns

S2-1：Construction of nanocone model

The open nanocone can be modeled as a wrapped graphene-like sheet. To have strain-free,

seamless wrapping, a sector must be cut out of the sheet. That sector should have an angle of $n \times 60$, where n = 1–5 and thus, the disclination angle has only certain discrete values $\theta = 60°, 120°, 180°, 240°, 300°$. **Figure S1** shows the modeling of nanocones of different disclination angles with the same height of 2 nm.

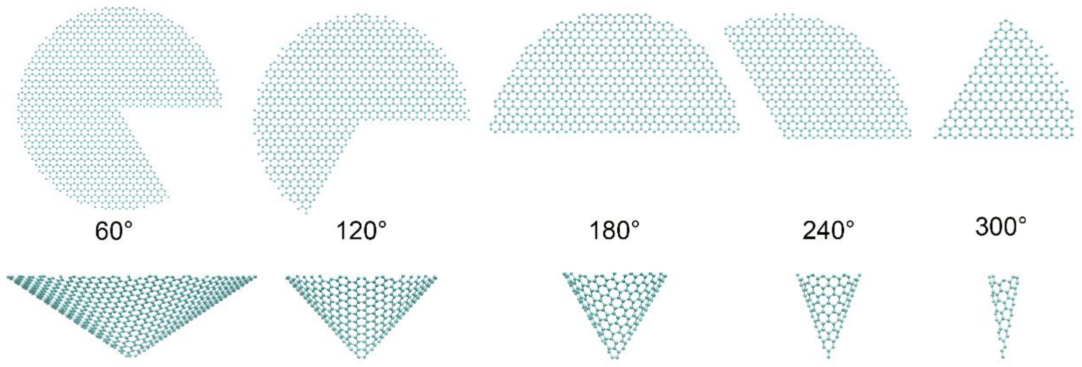

Figure S1 Nanocone models with different disclination angle

S3-1：Nanotube models with different curvatures, and the corresponding free energy barrier to pull 2OVN away from the nanotube.

To explore the relationship between nanotube curvature and the interaction between nanotubes and drugs. We selected four armchair nanotubes, (14,14) (18,18) (22,22) (26,26), with corresponding radius R 9.49 Å, 12.2 0Å, 14.92 Å, 17.63 Å for graphene, 9.69 Å, 12.46 Å, 15.24 Å, 18.00 Å for boron nitride. The atom number of the half tube here is the same for different curvatures, to eliminate the size effect.

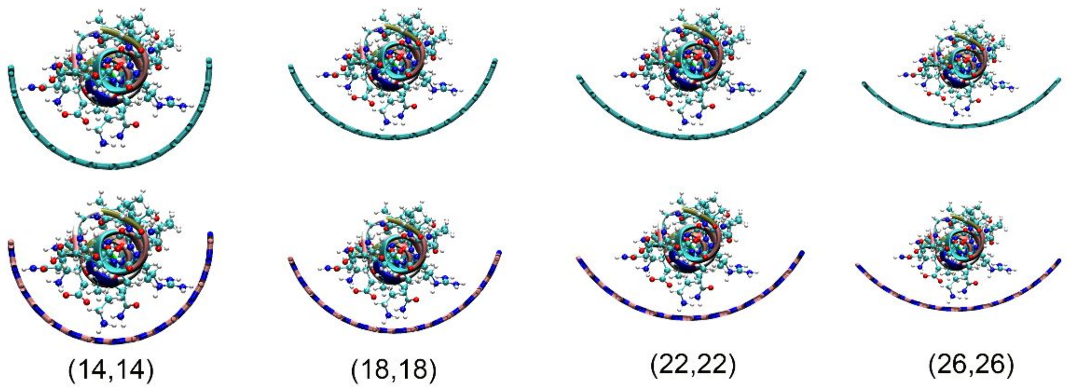

Figure S2 Initial configurations of half tubes made of graphene or boron nitride at different curvatures

Table S2 The free energy barrier for pulling a 2OVN molecule away from surfaces of C or BN with different diameter

	(14,14)	(18,18)	(22,22)	(26,26)
Radius R_C(Å)	9.49	12.20	14.92	17.63
Radius R_{BN}(Å)	9.69	12.46	15.24	18.00
ΔG_C (kcal/mol)	33.6	28.3	24.9	22.4
ΔG_{BN} (kcal/mol)	42.8	34.6	31.4	28.0

S4-1：Reverse drug delivery from tube to cone

Inspired by the delivery uncertainty of the BN_V-$C_{||}$ model, we simulated reverse drug delivery from tube to cone. In **Figure 5(c)**, for the blue triangle BN_V-$C_{||}$ model, ΔG (4.48 kcal·mol^{-1}) is positive. So, the delivery process from cone to tube is energetically unfavorable, obviously the delivery from tube to cone should be energetically favorable. We simulated the delivery of DOX at BN_V-$C_{||}$ (α=180°, β=6.78) model, by placing DOX in the center of the tube at the initial moment. The results are shown in **Figure S3**, DOX first reaches the junction quickly, stays for some time, and then is released to the cone, realizing the delivery from tube to cone. Therefore, we can achieve reverse drug delivery from the tube to the cone by using the

cone-dominated BN_V-$C_{||}$ model, which means we can freely adjust the position of the cone on the tube to deliver the drug upward or downward.

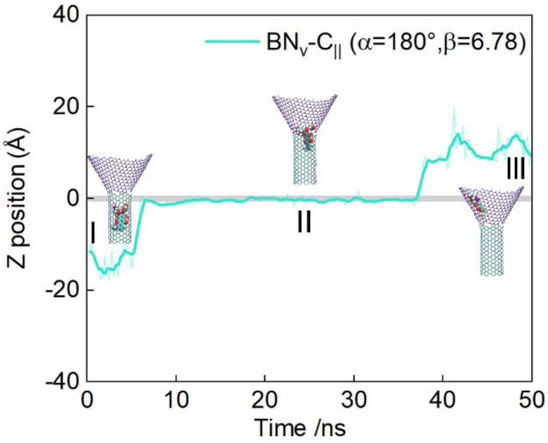

Figure S3 The position of the CoM of DOX on the Z axis as a function of simulated time. The snapshots Ⅰ, Ⅱ, Ⅲ correspond to the positions of DOX at the initial, junction, and final moments.

REFERENCES

(1) Melchor, S.; Dobado, J. A., CoNTub: An Algorithm for Connecting Two Arbitrary Carbon Nanotubes. *Journal of Chemical Information & Computer Sciences* **2004,** *44*, 1639-1646.

(2) Steinmetz, M. O.; Jelesarov, I.; Matousek, W. M.; Honnappa, S.; Jahnke, W.; Missimer, J. H.; Frank, S.; Alexandrescu, A. T.; Kammerer, R. A., Molecular basis of coiled-coil formation. *Proceedings of the National Academy of Sciences* **2007,** *104*, 7062-7067.

(3) Thakur, N.; Qureshi, A.; Kumar, M., VIRsiRNAdb: a curated database of experimentally validated viral siRNA/shRNA. *Nucleic Acids Res.* **2012,** *40*, D230-D236.

(4) Ansar, M.; Ashfaq, U. A.; Shahid, I.; Sarwar, M. T.; Javed, T.; Rehman, S.; Hassan, S.; Riazuddin, S., Inhibition of full length hepatitis C virus particles of 1a genotype through small interference RNA. *Virology Journal* **2011,** *8*, 1-6.

(5) Abraham, M. J.; Murtola, T.; Schulz, R.; Páll, S.; Smith, J. C.; Hess, B.; Lindahl, E., GROMACS: High performance molecular simulations through multi-level parallelism from laptops to supercomputers. *SoftwareX* **2015,** *1*, 19-25.

(6) Best, R. B.; Zhu, X.; Shim, J.; Lopes, P. E.; Mittal, J.; Feig, M.; MacKerell Jr, A. D., Optimization of the additive CHARMM all-atom protein force field targeting improved sampling of the backbone ϕ, ψ and side-chain $\chi1$ and $\chi2$ dihedral angles. *J. Chem. Theory Comput.* **2012,** *8*, 3257-3273.

(7) Hess, B.; van der Vegt, N. F., Hydration thermodynamic properties of amino acid analogues: a systematic comparison of biomolecular force fields and water models. *The journal of physical chemistry B* **2006,** *110*, 17616-17626.

(8) Tu, Y.; Lv, M.; Xiu, P.; Huynh, T.; Zhang, M.; Castelli, M.; Liu, Z.; Huang, Q.; Fan, C.; Fang, H., Destructive extraction of phospholipids from Escherichia coli membranes by graphene nanosheets. *Nat. Nanotechnol.* **2013,** *8*, 594-601.

(9) Luan, B.; Zhou, R., Atomic-scale fluidic diodes based on triangular nanopores in bilayer hexagonal boron nitride. *Nano Lett.* **2019,** *19*, 977-982.

(10) Zoete, V.; Cuendet, M. A.; Grosdidier, A.; Michielin, O., SwissParam: a fast force field generation tool for small organic molecules. *J. Comput. Chem.* **2011,** *32*, 2359-2368.

(11) Berger, O.; Edholm, O.; Jähnig, F., Molecular dynamics simulations of a fluid bilayer of dipalmitoylphosphatidylcholine at full hydration, constant pressure, and constant temperature. *Biophys. J.* **1997,** *72*, 2002-2013.

(12) Anezo, C.; de Vries, A. H.; Höltje, H.-D.; Tieleman, D. P.; Marrink, S.-J., Methodological issues in lipid bilayer simulations. *The Journal of Physical Chemistry B* **2003,** *107*, 9424-9433.

(13) Benz, R. W.; Castro-Román, F.; Tobias, D. J.; White, S. H., Experimental validation of molecular dynamics simulations of lipid bilayers: a new approach. *Biophys. J.* **2005,** *88*, 805-817.

(14) Kandt, C.; Ash, W. L.; Tieleman, D. P., Setting up and running molecular dynamics simulations of membrane proteins. *Methods* **2007,** *41*, 475-488.

(15) Hess, B.; Bekker, H.; Berendsen, H. J.; Fraaije, J. G., LINCS: A linear constraint solver for molecular simulations. *J. Comput. Chem.* **1997,** *18*, 1463-1472.

(16) Essmann, U.; Perera, L.; Berkowitz, M. L.; Darden, T.; Lee, H.; Pedersen, L. G., A smooth particle mesh Ewald method. *The Journal of chemical physics* **1995,** *103*, 8577-8593.

(17) Darden, T.; York, D.; Pedersen, L., Particle mesh Ewald: An N· log (N) method for Ewald sums in large systems. *The Journal of chemical physics* **1993,** *98*, 10089-10092.

(18) Bussi, G.; Donadio, D.; Parrinello, M., Canonical sampling through velocity rescaling. *The Journal of chemical physics* **2007,** *126*.

(19) Nosé, S.; Klein, M., Constant pressure molecular dynamics for molecular systems. *Mol. Phys.* **1983,** *50*, 1055-1076.

(20) Torrie, G. M.; Valleau, J. P., Nonphysical sampling distributions in Monte Carlo free-energy estimation: Umbrella sampling. *J. Comput. Phys.* **1977,** *23*, 187-199.

(21) Roux, B., The calculation of the potential of mean force using computer simulations. *Comput. Phys. Commun.* **1995,** *91*, 275-282.

(22) Kumar, S.; Rosenberg, J. M.; Bouzida, D.; Swendsen, R. H.; Kollman, P. A., Multidimensional free-energy calculations using the weighted histogram analysis method. *J. Comput. Chem.* **1995,** *16*, 1339-1350.

(23) Kumar, S.; Rosenberg, J. M.; Bouzida, D.; Swendsen, R. H.; Kollman, P. A., The weighted histogram analysis method for free-energy calculations on biomolecules. I. The method. *J. Comput. Chem.* **1992,** *13*, 1011-1021.
